# Supplementary material for: Phenotypic Characterization of Recombinant Marek’s Disease Virus in Live Birds Validates Polymorphisms Associated with Virulence
Source: Viruses. 2023 Nov 16;15(11):2263. doi: 10.3390/v15112263 (PMC10674313; doi:10.3390/v15112263)
Supplement: Supplementary file 1 [file viruses-15-02263-s001.zip › viruses-2660082-supplementary.pdf]

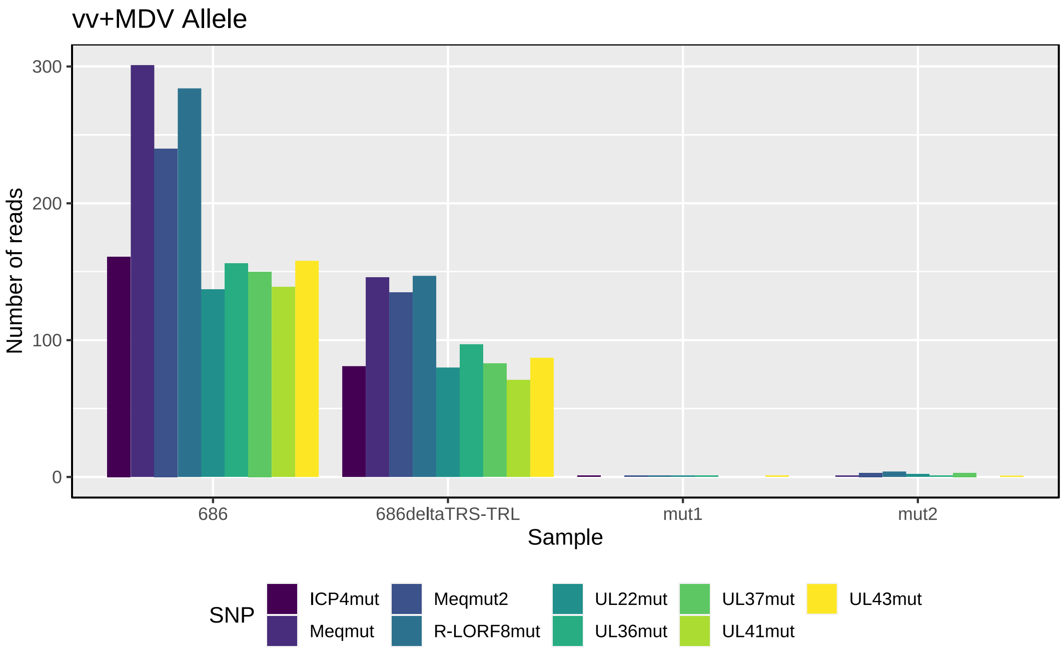

Figure S1. MiSeq sequence read depth for the vv+MDV allele in the 9 mutated nsSNP. The different colored bars denote the gene containing the mutated allele and the X axis identifies the BAC clone associated with the next generation sequencing reads.

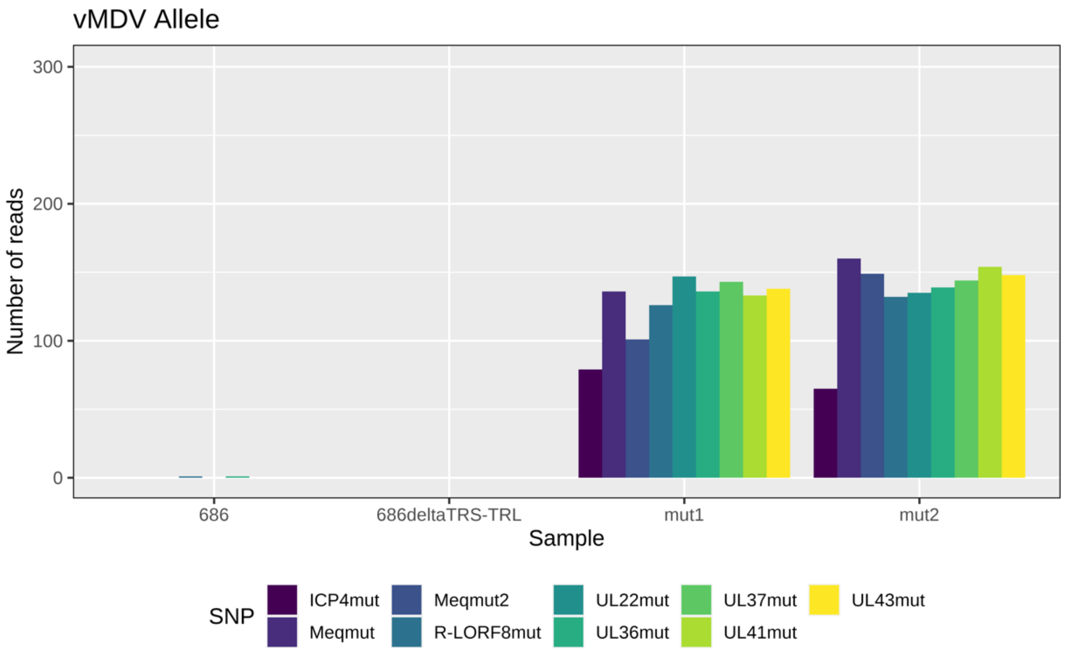

Figure S2. MiSeq sequence read depth for the vMDV allele in the 9 mutated nsSNP. The different colored bars denote the gene containing the mutated allele and the X axis identifies the BAC clone associated with the next generation sequencing reads.

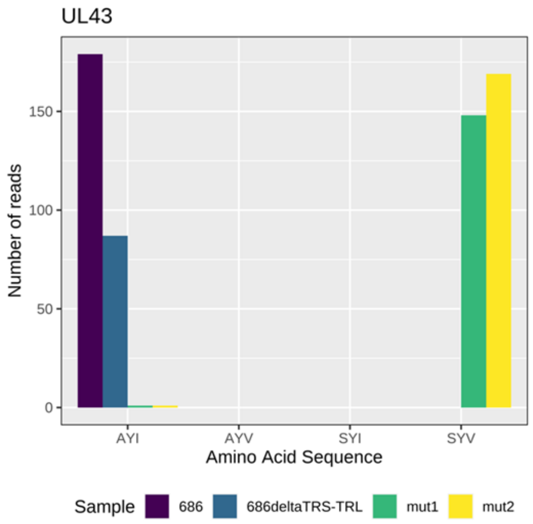

Figure S3. Read depth for the two non-synonymous SNPs identified in UL43. An G to T substitution upstream of the mutated allele on UL43 changes the amino acid sequence at this locus from A to S. The additional SNP segregated only in the mutated BAC clone with the mutated alleles coding as SYV at the protein sequence position 397-399.

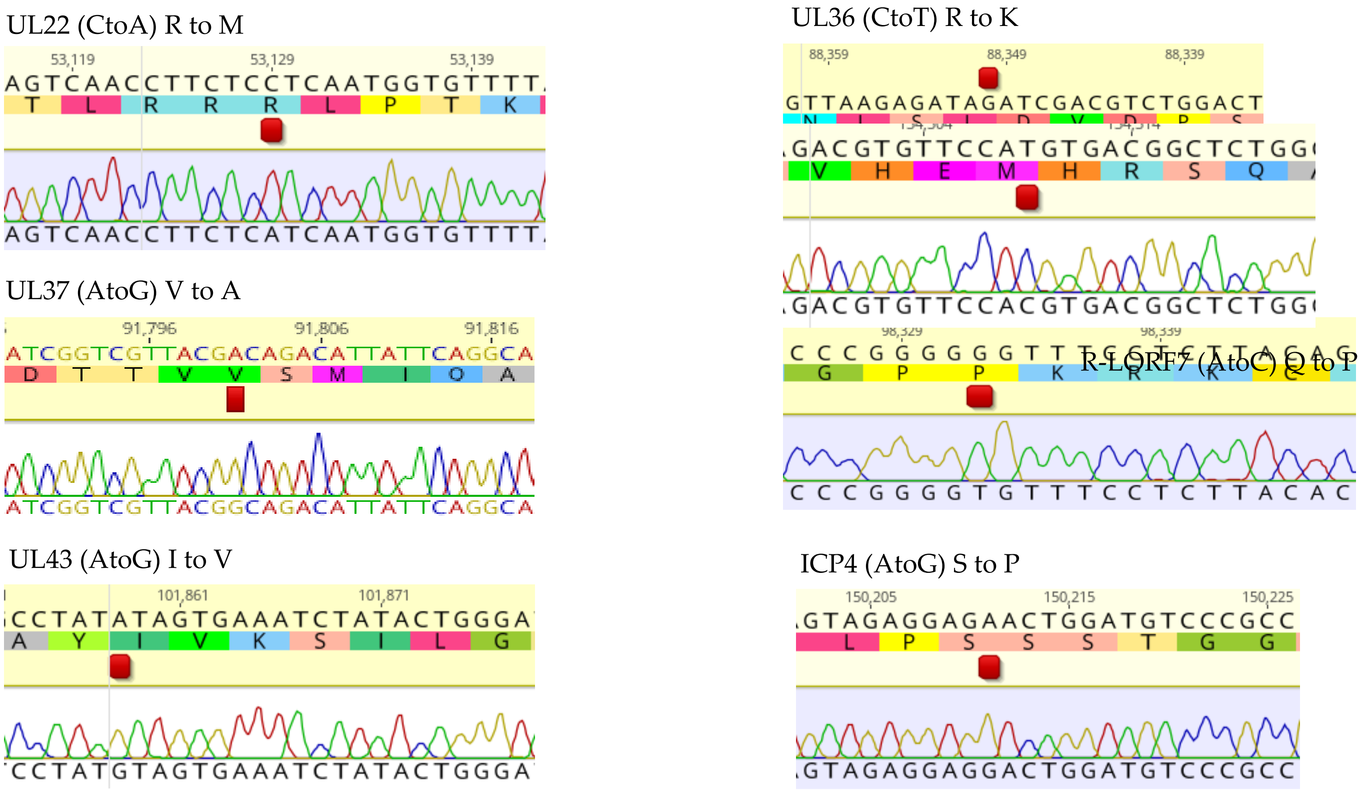

R-LORF7 (CtoT) R to C

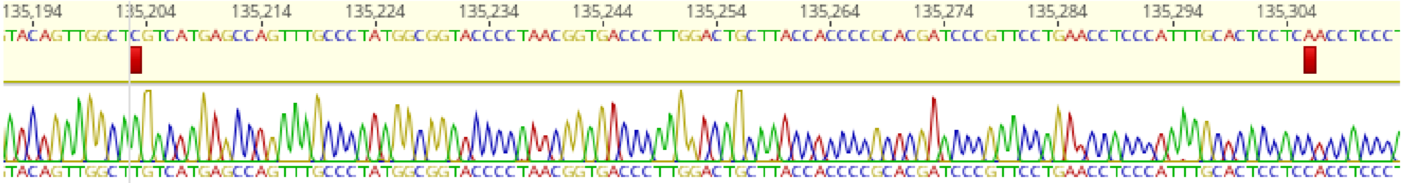

Figure S4. Sequence analysis of PCR amplicons from the SNPs modified 686-BAC virus. The alignment of sequencing data with parental 686-BAC was performed, and the specific changes in nucleotide sequences are indicated.

A)

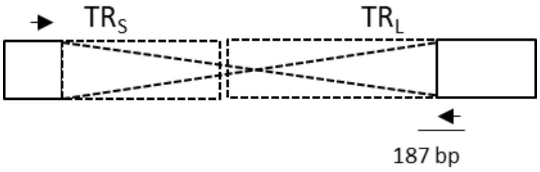

B)

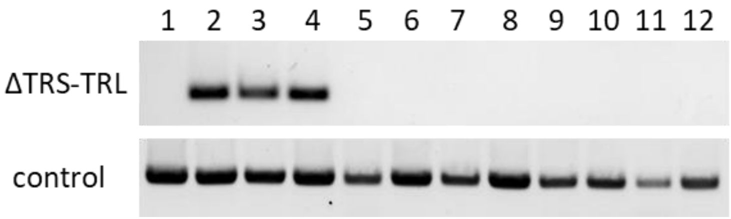

Figure S5. Restoration of TRS and TRL region of recombinant 686 viruses in virus infected cells and in vivo. A) The deleted region of MDV TRS and TRL are indicated, and primers used to amplify the junction of deleted regions are indicated. B) The junction of deletion was amplified from BAC plasmid DNA (lane 1-4), DNA extracted from virus infected CEFs at passage 7 (lane 5-8), and feather samples from virus infected chicken (lane 9-12). Lane 1,5,9: v686-BAC, Lane 2,6,10: v686ΔTRS-TRL, Lane 3,7,11: v686 mut1, Lane 4,8,12: v686 mut2. MDV UL22 region were amplified as a control with primers (Table S2).

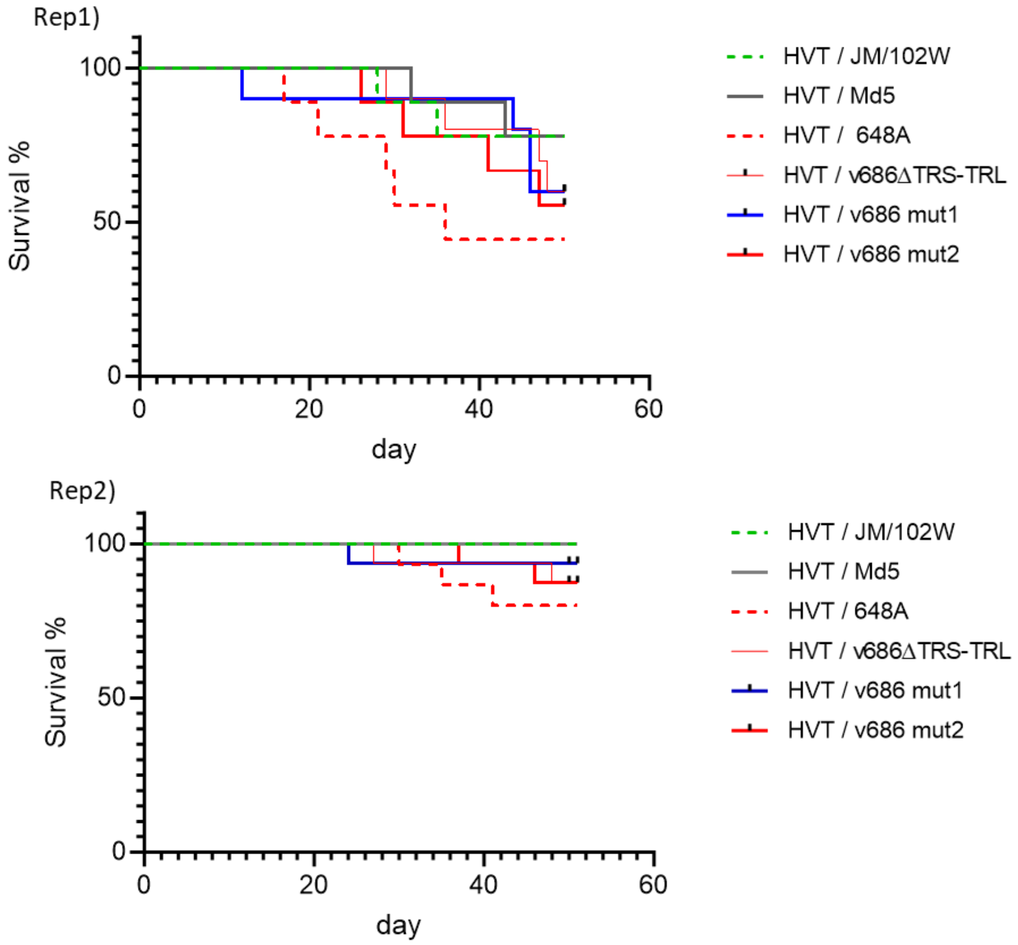

Figure S6. Survival curve of MDV JM/102W, Md5, 648A, v686ΔTRS-TRL, v686 mut1, or v686 mut2 challenged group in HVT-vaccinated birds.

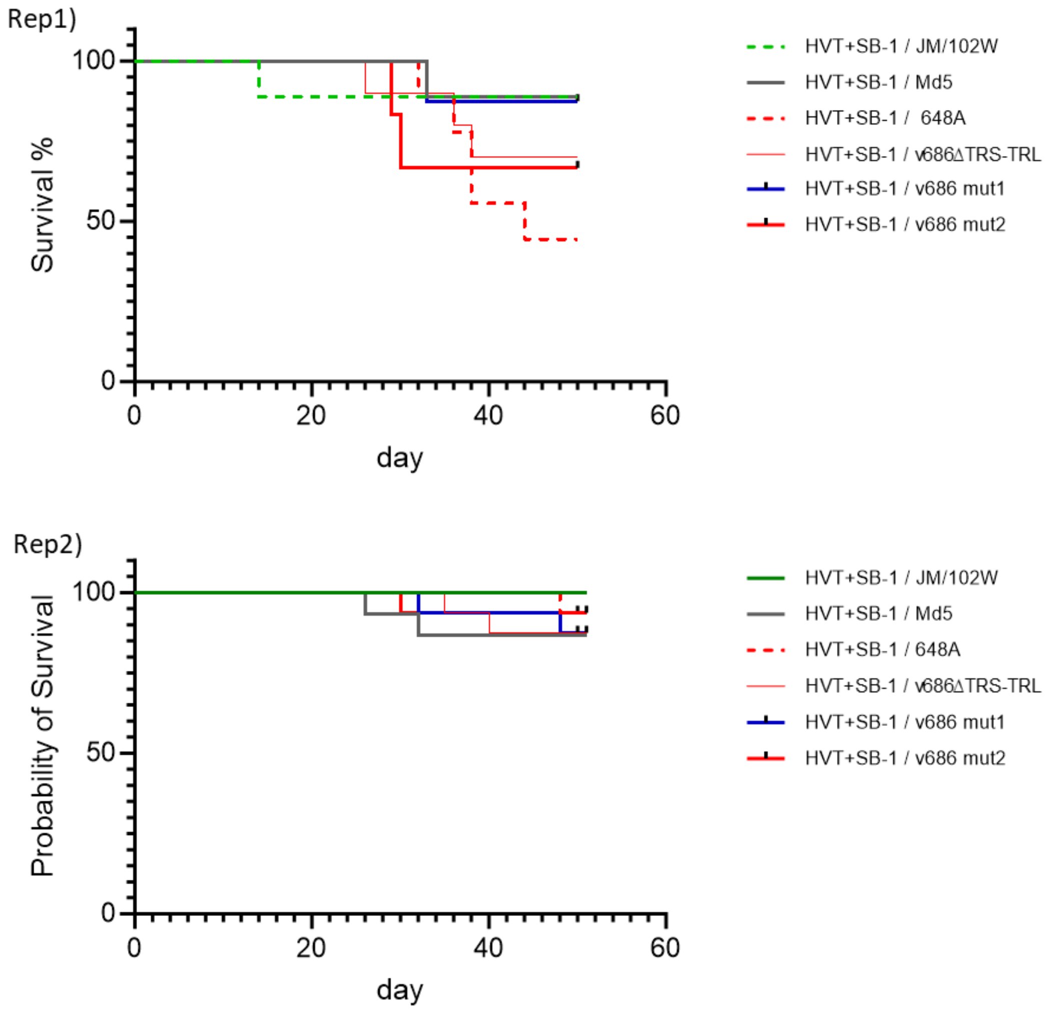

Figure S7. Survival curve of MDV JM/102W, Md5, 648A, v686ΔTRS-TRL, v686 mut1, or v686 mut2 challenged group in bivalent-vaccinated birds.

Table S1. The Oligonucleotides are used to delete the terminal repeat region in Marek's disease virus strain 686 genome.

Primer	Sequences (5′ to 3′)*
delTRS.FP	ATGGACAACCCGCCTGATTTCGACAGCTTGTTGGCAGCTTTTGACGAAGAgtgtaggctg-gagctgcttc
delTRL.RP	ACGTGTGTATCGTGGTCGTCTACTGTTTGTGGTGGTATTGAAACATCTTCaattgg-gatccgtcgacctg

*The upper case indicates the homologous sequences for MDV 686-BAC for recombination. The low case indicates that the sequences were used to amplify the selection of kanamycin-resistant gene cassette from pRL128.

Table S2. Oligonucleotides to amplify the region of SNPs modification in the genome of Marek's disease virus strain 686.

ORF	Primer	Sequence (5′ to 3′)	Amplicon (bp)
UL22	686UL22.FP	CCGTACGATTGTACCATTTGGG	944
	686UL22.RP	ATCTGGGGGAGCATGTTTCG	
UL36	686UL36.FP	ACGCCAACCACAATTAAACAA	996
	686UL36.RP	GCTCCAGATGATGTAGATCTGACT	
UL37	686UL37.FP	CGGCCAGATCCCCTACATTAA	991
	686UL37.RP	CCGTTCAGCGTGTCAGGATA	
UL41	686UL41.FP	CGCCGATCTTGCTCTTCGTA	897
	686UL41.RP	AAAAACTGCGAGGGTCATCA	
UL43	686UL43.FP	GCAACGCATGAAGAAGCCAT	886
	686UL43.RP	CTGTGGAAGGTGCATGAGGT	
R-LORF8	686R-LORF8.FP	GATAATGCCCTCGCCGATCA	991
	686R-LORF8.RP	TTTACACCTGTACCGTGCCC	
R-LORF7	686R-LORF7.FP	GATTCCTAGGCAGGCGTCTC	945
	686R-LORF7.RP	ATGTGGGGGAGATGGGGTAA	
ICP4	686ICP4.FP	GGAGATGGAATGGGAGCAGG	947
	686ICP4.RP	ATGGACAACCCGCCTGATTT	

Table S3. Marek's disease lesion score and tumor formation of ADOL 15 x 7 SPF chickens infected with recombinant MDV with SNPs modification and parental virus.

Rep1

Virus	# of birds	Chick mortality	Birds at risk	Thymus atrophy (TA)			Bursa atrophy (BA)			Nerve enlargement			
				# of birds	Lesion scores	Average	# of birds	Lesion scores	Average	# of birds	vagus	bra-chial	sciatic
None	15	0	15	0	0	0.00	0	0	0.0	0	0	0	0
v686-BAC	15	0	15	10	3,3,2,4,3,3,4,3,3,3,	3.10	10	3,2,3,3,3,3,3,3,3,2	2.8	3	3	2	2
v686ΔTRS-TRL	15	3	12	11	4,4,4,3,4,4,4,3,3,1,4	3.45	10	3,2,2,2,3,2,3,3,3,4	2.8	7	7	6	6
v686 mut1	15	7	8	7	2,3,4,4,4,2,3	3.14	7	2,3,4,3,4,2,3	3.0	2	2	2	1
v686 mut2	15	1	14	11	3,2,1,2,2,3,3,3,3,4,2	2.55	11	3,3,1,2,1,3,3,4,3,4,2	2.6	7	7	3	4

Virus	Birds at risk	# of birds with tumor	# of tumor per bird	Organ with tumor						
				gonad	spleen	kidney	heart	liver	pancreas	lung
None	15	0	0	0	0	0	0	0	0	0
v686-BAC	15	2	4,2	2	2	1	1	0	0	0
v686ΔTRS-TRL	12	6	1,2,1,1,2,3	5	2	0	2	1	0	0
v686 mut1	8	3	2,4,2,3	4	4	0	2	1	0	0
v686 mut2	14	9	4,3,2,7,1,1,1,4,3	3	7	2	7	4	1	2

Rep2

Virus	# of birds	Chick mortality	Birds at risk	Thymus atrophy (TA)			Bursa atrophy (BA)			Nerve enlargement			
				# of birds	Lesion scores	Average	# of birds	Lesion scores	Average	# of birds	vagus	brachial	sciatic
None	15	3	12	0	0	0.00	0	0	0.00	0	0	0	0
v686-BAC	15	6	9	9	4,3,3,4,4,3,3,3,3,	3.33	9	4,3,3,4,4,3,3,3,2	3.22	7	6	7	6
v686ΔTRS-TRL	15	7	8	8	3,4,4,3,4,4,2,3	3.38	8	3,4,4,3,4,4,2,3	3.44	4	4	4	3
v686 mut1	15	1	14	10	2,3,3,3,2,3,3,3,3,3	2.80	10	2,3,3,3,2,3,3,3,2,2,	2.60	6	6	4	2
v686 mut2	15	0	15	10	3,1,3,3,3,3,3,3,4,3	2.90	9	3,3,3,3,3,3,2,4,2	2.91	7	7	5	7

Virus	Birds at risk	# of birds with tumor	# of tumor per bird	Organ with tumor						
				proventricular	gonad	spleen	kidney	heart	liver	lung
None	12	0	0	0	0	0	0	0	0	0
v686-BAC	9	6	1,2,3,2,5,1	0	3	4	0	5	2	0
v686ΔTRS-TRL	8	3	4,2,2	0	2	2	0	2	2	0
v686 mut1	14	11	2,2,2,1,5,3,2,1,4,4,3	1	5	10	1	6	6	0
v686 mut2	15	9	4,3,2,7,1,1,1,4,3	2	7	7	2	7	5	1

Table S4. Marek's disease lesion score and tumor formation of commercial SPF chickens infected with recombinant MDV with SNPs modification and parental virus..

Rep1

Vaccine	Challenge	# of birds	Chick mortality	Birds at risk	Thymus atrophy (TA)			Bursa atrophy (BA)			Nerve enlargement			
					# of birds	Lesion scores	Average	# of birds	Lesion scores	Average	# of birds	vagus	bra-chial	sciatic
None	JM/102W	16	0	16	11	2,2,3,2,2,2,2,3,3,3,1	1.56	11	2,2,3,2,2,2,2,2,2,2,1	1.38	15	15	15	15
HVT		9	0	9	2	2,3	0.56	2	1,2	0.33	2	2	2	2
HVT+SB1		9	0	9	1	2	0.22	1	1	0.11	1	1	1	0
None	Md5	16	0	16	14	3,2,3,3,3,3,3,3,1,3,2,3,2,3	2.31	15	3,3,2,3,3,2,3,2,3,1,2,1,3,2,3	2.25	14	14	14	13
HVT		9	0	9	3	2,3,2	0.78	3	2,2,1	0.56	3	2	2	3
HVT+SB1		9	0	9	1	3	0.33	1	3	0.33	2	2	2	2
None	648A	16	0	16	16	3,3,3,3,2,3,3,3,3,3,3,3,2,3,3,2	2.81	16	2,3,3,3,2,2,3,3,3,3,2,3,3,2,2,2	2.56	15	15	15	15
HVT		9	0	9	7	3,1,2,3,2,3,2	1.78	7	2,1,2,3,3,3,2	1.78	8	6	8	7
HVT+SB1		9	0	9	5	2,2,3,3,3	1.44	5	1,1,3,2,2	1.00	5	5	5	5
None	v686ΔTRS-TRL	16	1	15	15	2,3,3,3,3,3,3,2,2,3,3,2,2,2,3	2.60	15	2,2,2,3,2,2,2,2,2,2,3,2,2,2,3	2.20	15	15	15	15
HVT		10	0	10	5	3,3,1,3,1	1.10	3	3,3,2	0.80	6	4	6	5
HVT+SB1		10	0	10	6	1,2,3,3,3,2,	1.40	7	1,2,3,3,1,3,2	1.50	7	6	7	6
None	v686 mut1	16	0	16	16	3,3,3,3,3,2,2,2,3,3,3,2,2,3,3	2.67	15	2,2,3,3,2,3,3,2,3,2,2,2,2,2,3	2.40	15	12	15	15
HVT		10	0	10	5	2,2,2,2,2	1.00	4	1,2,2,2	0.70	5	5	5	5
HVT+SB1		10	2	8	2	3,3	0.75	2	3,3	0.75	2	2	2	1
None	v686 mut2	16	0	16	16	3,2,1,3,3,3,3,3,3,3,3,3,3,3,2,2	2.69	16	3,2,2,3,2,3,2,3,3,2,3,2,2,2,2,1	2.38	16	14	16	16
HVT		10	1	9	4	3,2,2,1	0.89	4	3,2,2,1	0.89	4	4	4	4
HVT+SB1		10	4	6	2	3,2	0.83	3	2,2,2	1.00	3	3	3	3

Vaccine	Challenge	# of birds	# of bird with tumor	# of tumor per bird	Organs with tumor											
					proven-tricular	gonad	spleen	kidney	heart	liver	pancreas	intestine	lung	skin	ceca	bursa
None	JM/102W	16	5	2,1,1,2,1	0	1	1	1	4	0	0	0	0	0	0	0
HVT		9	1	1	0	1	0	0	0	0	0	0	0	0	0	0
HVT+SB1		9	0	0	0	0	0	0	0	0	0	0	0	0	0	0
None	Md5	16	11	1,1,1,2,1,1,1,1,1,2,1	0	0	2	0	10	1	0	0	0	0	0	0
HVT		9	3	1,2,3	0	0	2	0	2	2	0	0	0	0	0	0
HVT+SB1		9	2	1,1	0	0	0	0	2	0	0	0	0	0	0	0
None	648A	16	10	1,1,1,1,1,1,2,1,1,2	0	1	6	0	5	0	0	0	0	0	0	0
HVT		9	5	1,3,3,2,1	0	1	3	2	4	0	0	0	0	0	0	0
HVT+SB1		9	4	1,2,1,1	0	2	1	1	0	0	0	0	0	0	0	0
None	v686ΔTRS-TRL	15	6	1,1,1,1,1,1	0	1	2	0	2	1	0	0	0	0	0	0
HVT		10	2	1,1	0	0	0	0	1	0	0	1	0	0	0	0
HVT+SB1		10	3	5,2,1	1	1	0	0	3	0	0	1	1	1	0	0
None	v686 mut1	16	15	1,1,2,2,2,3,3,2,1,2,2,1,2,1	1	2	7	1	10	4	0	0	0	0	0	0
HVT		10	3	4,4,1	0	2	1	1	0	1	0	2	0	0	1	1
HVT+SB1		8	1	3	0	0	0	0	1	1	0	0	0	0	0	0
None	v686 mut2	16	13	3,1,3,5,2,2,1,2,2,4,1,1,2	0	4	10	2	6	3	2	2	0	0	0	0
HVT		9	3	2,4,1	0	1	2	1	2	1	0	0	0	0	0	0
HVT+SB1		6	1	1	0	1	0	0	0	0	0	0	0	0	0	0

Rep 2

Vaccine	Challenge	# of birds	Chick mortality	Birds at risk	Thymus atrophy (TA)			Bursa atrophy (BA)			Nerve enlargement			
					# of birds	Lesion scores	Average	# of birds	Lesion scores	Average	# of birds	vagus	bra-chial	sciatic
None	JM/102W	13	0	13	5	1,1,3,3,1	0.69	4	1,2,3,2	0.62	6	6	6	6
HVT		16	0	16	0		0.00	0		0.00	0			
HVT+SB1		16	0	16	0		0.00	0		0.00	0			
None	Md5	13	0	13	13	3,3,3,2,3,3,3,3,2,2,3,3,2	2.69	13	3,3,3,1,2,3,3,3,2,2,3,2,2	2.46	13	13	13	13
HVT		16	1	15	0		0.00	0		0.00	1	0	1	1
HVT+SB1		16	1	15	2	3,2	0.33	2	2,1	0.20	3	2	3	3
None	648A	13	2	11	13	3,3,3,3,3,2,3,3,2,3,3,2,2	3.18	11	3,3,3,2,3,3,4,3,3,4,3	3.09	11	11	11	11
HVT		16	1	15	8	2,1,3,3,1,1,1,2	0.93	8	1,2,2,2,2,1,2,1	0.87	10	10	10	10
HVT+SB1		16	0	16	2	3,3	0.31	3	1,2,2	0.31	4	4	4	4
None	v686ΔTRS-TRL	13	0	13	12	3,3,3,3,3,2,3,3,2,3,3,2,2	2.69	13	3,2,2,3,3,2,2,3,2,3,2,2,2	2.38	13	13	13	13
HVT		16	0	16	2	3,3	0.38	2	2,2	0.25	2	2	2	2
HVT+SB1		16	0	16	2	3,3	0.38	2	3,2	0.31	2	2	2	2
None	v686 mut1	13	0	13	9	3,3,3,3,3,3,3,3,2	2.00	9	2,3,2,2,3,3,2,2,2	1.62	9	9	9	9
HVT		16	0	16	3	2,2,2	0.38	3	2,2,2	0.38	3	3	3	2
HVT+SB1		16	0	16	2	3,1	0.25	1	3	0.19	2	1	2	2
None	v686 mut2	13	1	12	12	3,2,3,3,2,2,2,2,3,2,3	2.25	11	3,2,2,2,2,2,2,2,3,2,3	2.08	12	11	12	11
HVT		16	0	16	2	2,3	0.31	2	2,2	0.25	3	3	3	3
HVT+SB1		16	0	16	1	3	0.19	1	2	0.13	2	1	2	2

Vaccine	Challenge	# of birds	# of bird with tumor	# of tumor per bird	Organs with tumor								
					proven-tricular	gonad	spleen	kidney	heart	liver	pancreas	intestine	thymus
None	JM/102W	13	1	1	0	0	1	0	0	0	0	0	0
HVT		16	0	0	0	0	0	0	0	0	0	0	0
HVT+SB1		16	0	0	0	0	0	0	0	0	0	0	0
None	Md5	13	7	3,3,3,1,3,1,1	0	0	3	1	7	4	0	0	0
HVT		15	0	0	0	0	0	0	0	0	0	0	0
HVT+SB1		15	1	1	0	0	1	0	0	0	0	0	0
None	648A	11	6	2,2,1,1,1,1	1	0	2	0	5	0	0	0	0
HVT		15	4	1,1,2,1	0	0	4	0	1	0	0	0	0
HVT+SB1		16	2	3,1	0	0	2	0	1	1	0	0	0
None	v686ΔTRS-TRL	13	8	2,3,1,3,1,1,3,2	0	1	5	2	7	1	0	0	0
HVT		16	2	2,2	0	1	1	0	2	0	0	0	0
HVT+SB1		16	1	1	0	0	0	0	1	0	0	0	0
None	v686 mut1	13	7	2,2,3,1,1,3,2	0	1	4	0	5	4	0	0	0
HVT		16	2	1,3	0	1	1	0	1	0	0	1	0
HVT+SB1		16	2	1,1	0	1	0	0	1	0	0	0	0
None	v686 mut2	12	9	4,1,3,1,1,1,1,3,5	0	2	5	3	5	4	0	0	1
HVT		16	1	5	1	1	1	0	1	1	0	0	0
HVT+SB1		16	2	2,3	0	0	1	2	1	1	0	0	0

Table S5. Statistical differences of survival in pathotype assay

Rep1	none	HVT	HVT+SB-1	none	HVT	HVT+SB-1	none	HVT	HVT+SB-1	none	HVT	HVT+SB-1	none	HVT	HVT+SB-1
	Md5			648A			v686ΔTRS-TRL			v686 mut1			v686 mut2		
None / JM/102W	ns			**			**			**			**		
HVT / JM/102W		ns			ns			ns			ns			ns	
HVT+ SB-1 / JM/102W			ns			ns			ns			ns			ns
None / Md5				*			**			*			ns		
HVT / Md5					ns			ns			ns			ns	
HVT+ SB-1 / Md5						ns			ns			ns			ns
None / 648A							ns			ns			ns		
HVT / 648A								ns			ns			ns	
HVT+ SB-1 / 648A									ns			ns			ns
None / v686ΔTRS-TRL										**			**		
HVT / v686ΔTRS-TRL											ns			ns	
HVT+ SB-1/v686ΔTRS-TRL												ns			ns
None / v686 mut1													ns		
HVT / v686 mut1														ns	
HVT+ SB-1/ v686 mut1															ns

Rep2	none	HVT	HVT+SB-1	none	HVT	HVT+SB-1	none	HVT	HVT+SB-1	none	HVT	HVT+SB-1	none	HVT	HVT+SB-1
	Md5			648A			v686ΔTRS-TRL			v686 mut1			v686 mut2		
None / JM/102W	**			**			**			*			**		
HVT / JM/102W		ns			ns			ns			ns			ns	
HVT+ SB-1 / JM/102W			ns			ns			ns			ns			ns
None / Md5				ns			ns			ns			ns		
HVT / Md5					ns			ns			ns			ns	
HVT+ SB-1 / Md5						ns			ns			ns			ns
None / 648A							*			*			*		
HVT / 648A								ns			ns			ns	
HVT+ SB-1 / 648A									ns			ns			ns
None / v686ΔTRS-TRL										ns			ns		
HVT / v686ΔTRS-TRL											ns			ns	
HVT+ SB-1/v686ΔTRS-TRL												ns			ns
None / v686 mut1													ns		
HVT / v686 mut1														ns	
HVT+ SB-1/v686 mut1															ns

Statistical differences (p-value < 0.05) between groups are indicated (ns = not significant, *p-value < 0.05, ** p-value < 0.01).
